# Role of Family Planning in Women With Multiple Sclerosis in Switzerland: Results of the Women With Multiple Sclerosis Patient Survey

**DOI:** 10.3389/fneur.2018.00821

**Published:** 2018-10-10

**Authors:** Christian P. Kamm, Sarah Muehl, Dennis Mircsof, Stefanie Müller, Adam Czaplinski, Lutz Achtnichts, Petra Stellmes, Gabrielle Di Virgilio

**Affiliations:** ^1^Neurology and Neurorehabilitation Centre, Luzerner Kantonsspital, Lucerne, Switzerland; ^2^Department of Neurology, Inselspital, Bern University Hospital and University of Bern, Bern, Switzerland; ^3^Merck (Schweiz) AG, Zug, Switzerland, an affiliate of Merck KGaA, Darmstadt, Germany; ^4^Department of Neurology, Cantonal Hospital of St. Gallen, St. Gallen, Switzerland; ^5^Neurozentrum Bellevue AG, Zurich, Switzerland; ^6^Department of Neurology, Cantonal Hospital of Aarau, Aarau, Switzerland; ^7^Hôpital Riviera-Chablais, Vaud-Valais, Vevey, Switzerland

**Keywords:** pregnancy, family planning, multiple sclerosis, disease modifying drugs, treatment decision

## Abstract

**Background:** Women of child bearing age with multiple sclerosis (MS) must carefully consider treatments when planning a family, since disease modifying drugs (DMDs) are contraindicated during pregnancy.

**Objectives:** This questionnaire-based study aimed to improve understanding of the effect of family planning on treatment decisions in female, Swiss MS patients.

**Methods:** Female patients with MS (aged 18–55 years) participated in the 26-question survey between September 2014 and August 2015. Information captured included patient background, family planning status, treatment course, and previous pregnancies.

**Results:** In total, 271 questionnaires distributed from 15 MS centres were returned for analysis. Of these, 250 (92.3%) participants received DMD therapy and 106 (39.1%) wanted children or were pregnant. Significantly more patients with a short-term plan to conceive within 2 years were treated with injectables (19/54) compared with those without a plan to conceive (19/108; *p* = 0.013). A proportionally greater number of women not planning to conceive took oral (34/108) or infusion therapies (41/108) compared with those with a short- (13/54 and 16/54, respectively) or medium-term (after 2 years or more; infusion therapy only, 14/44) plan to conceive.

**Conclusion:** The study highlights that pregnancy remains an important yet unresolved concern in the treatment of MS patients. Nearly all women received DMD treatment, and type of DMD treatment was influenced by family planning, with significantly more women with a short-term plan to conceive using injectables.

## Introduction

In Switzerland, approximately 10,000 patients had MS in 2016 (1). The disease preferentially affects women and in recent decades, the ratio of women to men who develop the disease has risen (from 2.3:1 to 3.5:1) (2). MS is most often diagnosed between the ages of 20 and 40 years, therefore, many women with MS are of childbearing age and may plan to conceive (3).

Fertility and the course of pregnancy are not affected by MS (4). However, problems arise regarding the effective treatment of MS using disease modifying drugs (DMDs) in women who plan to become pregnant because none of the DMDs are officially approved for use during pregnancy in Switzerland. In clinical practice, treatment with some DMDs is stopped before pregnancy, some are given until pregnancy occurs, and some DMDs may be used during pregnancy after an individual risk-benefit analysis (5).

Thus far, studies suggest that use of injectable platform DMDs in early pregnancy has minimal effect on pregnancy outcomes (6–12). Recent registry data investigating the effects of interferon β (IFN β) exposure during early pregnancy showed there were no or minor differences in birth weight, birth length, premature birth, or other adverse pregnancy outcomes, compared with women with no DMD exposure (6). Similarly, glatiramer acetate (GA) has shown no known effects on pregnancy outcomes (7).

Data for newer treatments have also emerged on this topic. In a cohort of 355 pregnancies exposed early on to natalizumab, outcomes appeared unchanged except the major birth defect rate, which was higher than that observed in the general population. No specific pattern of birth defects was observed that would suggest a drug effect (13). In addition, some newborns, whose mothers were exposed to natalizumab during the third trimester of pregnancy, displayed haematologic alterations such as thrombocytopaenia and anaemia (14). A safety database with fingolimod that recorded 324 live births, found no increase in incidence of congenital malformations when compared with the general population (15). However, a smaller study that reported birth outcomes from 41 pregnancies, suggested fingolimod exposure during pregnancy was associated with a higher occurrence of foetal abnormalities, which included a case of acrania and tetralogy of Fallot (16). Interestingly, teriflunomide was also shown to have no effect on the occurrence of birth defects in 83 pregnancies (17), despite this drug causing reproductive toxicity in animals (18). However, the statistical power of this study was not sufficient to establish absolute teratogenicity (17), a factor that should be considered in all studies that evaluate drug effects on pregnancy outcomes (19).

Since treatment choice may be influenced by pregnancy plans, women with MS must weigh the possibility of a pregnancy during treatment, against the risk of disease progression without MS treatment when planning to conceive (5). Here we present the Swiss Women with MS patient survey, which as the first of its kind, aimed to improve the understanding of the effect of family planning on treatment decisions in women with MS of child bearing age, the influencing factors, and how the subject is addressed in the context of their treatment.

## Methods

### Patients

Between September 2014 and August 2015, the Women with MS patient survey was conducted in Switzerland across 15 specialised MS centres. To obtain a cohort that best represented the Swiss MS population, eight centres were hospital-based and seven were private practices. Inclusion criteria were female patients with McDonald MS 2010 (20), aged 18–55 years. Child-bearing age was categorised as aged between 18–45 years; women aged >45–55 years were included, given that part of this survey was retrospective (see below, questions 19–26). Patient inclusion was also independent of disease course, DMD treatment and family planning status. The main exclusion criteria were any diseases or conditions that would affect the adequate performance of the study procedures.

### Questionnaire

The questionnaire was designed by an independent company (Appletree CI Group AG [ACG+], Winterthur, Switzerland) for paper and online data collection in German, French and Italian languages. Printed questionnaires were transmitted to the MS centres in sealed envelopes; each had a unique study number that was associated with an MS centre to facilitate questionnaire tracking. The questionnaires were distributed to eligible patients during routine appointments at the MS centres. Questionnaires answered on paper were sent directly to ACG+ in post-paid envelopes. Alternatively, patients could answer questionnaires online using a unique code that was printed on the questionnaire envelopes. The exact return rate was not evaluated and reminders were not sent due to the anonymised nature of the questionnaire. The patient survey contained 26 multiple choice questions that were answered anonymously. Questions 1–7 determined the participants' background and circumstances. The disease course of each participant was provided by their neurologists (question 5). Questions 8–18 established the treatment course, disease state and family planning status of the participants. The remaining questions (19–26) captured information regarding previous pregnancies. In order to evaluate comprehensibility, the questionnaire was given to 10 MS patients prior to commencing the study, however no major adaptions were required. The questionnaire was not validated before the study was initiated. The final version of the questionnaire in English is shown as Supplementary Material.

The study design was discussed with the Ethics Committee of Bern, Switzerland, and a decision was made that no written patient information or patient consent forms were required given that the questionnaire was anonymised.

### Statistical analysis

Key variables in this study, upon which the analysis was based, included the patients' family planning status (question 8) and treatment choice and pregnancy (question 12). Patients were stratified into groups according to their plans to conceive, which included no plan, short-term plan (i.e., planning to conceive within 2 year) and medium-term plan (i.e., planning to conceive after 2 years or more). For the analysis, DMD treatments were categorised into the following groups: injectables (subcutaneous [sc] interferon β-1a [sc IFN β-1a], intramuscular [im] IFN β-1a, sc IFN β-1b and glatiramer acetate [GA]), infusion (natalizumab), oral (fingolimod and teriflunomide) and “other” therapies. Dimethyl fumarate (DMF) was not specifically included because it was approved in Switzerland near the end of 2014 and therefore was not significant to this survey, since no participants were using the drug. Further key variables included age category (< 20, 20–30, 31–40, 41–45, >45 years), time since diagnosis (0–3, 3–5, 5–10, >10 years), treatment with DMD (yes or no) and importance of therapy in regards to pregnancy (very important, important or not important).

The questionnaire data were mostly analysed descriptively. However, the responses to question 10 were analysed by applying the Pearson's chi-squared test on the respective contingency tables. The test was performed in SPSS (SPSS 22 under the operating system Windows 7 Professional, Service Pack 1) to determine statistical differences between groups (significance level 0.05, 2-sided). The survey sample size of 271 was sufficient to represent a population of ≤ 100,000 with a confidence interval of ±5% on a confidence level of 90%.

## Results

### Patient characteristics

In total, 271 questionnaires were returned for analysis (252 questionnaires were answered on paper; and 19 online). Of these, 177 (63.5%) questionnaires were answered by patients treated in hospital and 94 (34.7%) questionnaires by patients treated by practice based neurologists. Within the study population, 79.3% (215/271) of patients were of childbearing age (18–45 years) and the majority (91.9%; 249/271) were diagnosed with relapsing remitting multiple sclerosis (RRMS). In addition, 39.1% (106/271) were pregnant or wanted a child (Table 1). For those of childbearing age (18–45 years), 48.4% (104/215) wanted children or were pregnant, whereas 38.1% (82/215) had no plan to conceive and 13.5% (29/215) did not answer the question (see below, Table 5).

**Table 1:** Patient characteristics in the study population (*N* = 271).

**Parameter**	***n* (%)**
**AGE DISTRIBUTION, YEARS**	
< 20	3 (1.1)
20–30	77 (28.4)
31–40	97 (35.8)
41–45	38 (14.0)
>45	52 (19.2)
Unspecified	4 (1.5)
**DIAGNOSIS**	
CIS	7 (2.6)
RRMS	249 (91.9)
SPMS	6 (2.2)
Unspecified	9 (3.3)
**PLAN TO CONCEIVE**	
No plan	117 (43.2)
Currently pregnant	4 (1.5)
Short-term	58 (21.4)
Medium-term	44 (16.2)
Unspecified	48 (17.7)
**DMD TREATMENT**	
Yes	250 (92.3)
No	21 (7.7)
**DMD TREATMENT (*****n*** = **250)**	
im IFN β-1a	8 (3.2)
sc IFN β-1a	13 (5.2)
sc IFN β-1b	16 (6.4)
Fingolimod	71 (28.4)
GA	17 (6.8)
Natalizumab	88 (35.2)
Teriflunomide	5 (2.0)
“Other”	32 (12.8)

*n, number; CIS, clinically isolated syndrome; RRMS, relapsing remitting multiple sclerosis; SPMS, secondary progressive multiple sclerosis; DMD, disease modifying drug; im, intramuscular; sc, subcutaneous; IFN, interferon; GA, glatiramer acetate*.

The majority of survey participants were receiving DMD treatment (92.3%, 250/271). Of those receiving treatment, 21.6% (54/250) were taking injectable therapies (sc IFN β-1a [5.2%, 13/250], im IFN β-1a [3.2%, 8/250], sc IFN β-1b [6.4%, 16/250] and GA [6.8%, 17/250]), 30.4% (76/250) oral therapies (fingolimod [28.4%, 71/250] and teriflunomide [2%, 5/250]), 35.2% (88/250) infusion therapy (natalizumab), and 12.8% (32/250) were using “other” treatments.

The importance of specific factors in context of the disease were scored by patients on a scale of 1–5 (where 1 = not very important and 5 = very important). For participants in the whole population or planning to conceive, there was little or no difference in the mean score (± standard deviation) for delay in disability (total population, 4.8 [± 0.73]; women planning to conceive, 4.8 [± 0.61]), independence (4.8 [± 0.66]; 4.8 [± 0.59]), relapse free (4.7 [± 0.73]; 4.8 [± 0.60]), partnership (4.4 [± 1.02]; 4.5 [± 0.81]) and work (4.1 [± 1.04]; 4.2 [± 0.88]). Family planning was more important to women planning to conceive (4.3 [± 0.83]), compared with the whole population (3.2 [± 1.61]).

Baseline characteristics of those patients planning to conceive (short- [within 2 years] or long-term [after two 2 years or more]) are shown according to age group in Table 2. These data include the type of MS, choice of DMD and importance of treatment in relation to pregnancy.

**Table 2:** Characteristics of patients planning to conceive in accordance with age.

	**Age, years, distribution, n (%)**						
	** < 20**	**20–30**	**31–40**	**41–50**	**>45**	**Unspecified**	**Total**
	***n* = 2**	***n* = 63**	***n* = 32**	***n* = 3**	***n* = 1**	***n* = 1**	***n* = 102**
**DIAGNOSIS**							
CIS	1 (50)	3 (4.8)	1 (3.1)	0 (0.0)	0 (0.0)	0 (0.0)	5 (4.9)
RRMS	1 (50)	60 (95)	30 (94)	2 (67)	1 (100)	1 (100)	95 (93)
SPMS	0 (0.0)	0 (0.0)	0 (0.0)	0 (0.0)	0 (0.0)	0 (0.0)	0 (0.0)
Unspecified	0 (0.0)	0 (0.0)	1 (3.1)	1 (33)	0 (0.0)	0 (0.0)	2 (2.0)
**CHOICE OF DMD**							
im IFN β-1a	0 (0.0)	2 (3.2)	1 (3.1)	0 (0.0)	0 (0.0)	0 (0.0)	3 (2.9)
sc IFN β-1a	0 (0.0)	4 (6.3)	5 (16)	1 (33)	0 (0.0)	0 (0.0)	10 (9.8)
sc IFN β-1b	0 (0.0)	3 (4.8)	0 (0.0)	0 (0.0)	1 (100)	0 (0.0)	4 (3.9)
Fingolimod	0 (0.0)	18 (29)	8 (25)	1 (33)	0 (0.0)	1 (100)	28 (27)
GA	1 (50)	6 (9.5)	4 (12)	0 (0.0)	0 (0.0)	0 (0.0)	11 (11)
Natalizumab	0 (0.0)	20 (32)	9 (28)	1 (33)	0 (0.0)	0 (0.0)	30 (29)
Other	1 (50)	8 (13)	3 (9.4)	0 (0.0)	0 (0.0)	0 (0.0)	12 (12)
No therapy	0 (0.0)	2 (3.2)	2 (6.3)	0 (0.0)	0 (0.0)	0 (0.0)	4 (3.9)
**IMPORTANCE OF TREATMENT IN RESPECT TO PREGNANCY**							
Very important	1 (50)	13 (21)	9 (28)	0 (0.0)	1 (100)	0 (0.0)	24 (24)
Important	0 (0.0)	19 (30)	10 (31)	0 (0.0)	0 (0.0)	1 (100)	30 (29)
Not important	1 (50)	31 (49)	12 (38)	3 (100)	0 (0.0)	0 (0.0)	47 (46)
Unspecified	0 (0.0)	0 (0.0)	1 (3.1)	0 (0.0)	0 (0.0)	0 (0.0)	1 (1.0)
**FREQUENCY OF DISCUSSING FAMILY PLANNING WITH NEUROLOGIST**							
At every consultation	1 (50)	22 (35)	5 (16)	0 (0.0)	0 (0.0)	0 (0.0)	28 (27)
Sporadically	0 (0.0)	21 (33)	14 (44)	1 (33)	0 (0.0)	0 (0.0)	36 (35)
Only when asked by patient	0 (0.0)	17 (27)	12 (38)	1 (33)	1 (100)	1 (100)	32 (31)
Never	1 (50)	3 (4.8)	1 (3.1)	1 (33)	0 (0.0)	0 (0.0)	6 (5.9)
**HOW DID THEY LEARN OF OPTIONS?**							
Treating neurologist	1 (50)	45 (71)	28 (88)	1 (33)	1 (100)	1 (100)	77 (75)
GP	0 (0.0)	3 (4.8)	0 (0.0)	0 (0.0)	1 (100)	0 (0.0)	4 (3.9)
MS-Nurse	2 (100)	8 (13)	4 (12)	0 (0.0)	1 (100)	0 (0.0)	15 (15)
Gynaecologist	0 (0.0)	8 (13)	4 (12)	0 (0.0)	1 (100)	1 (100)	14 (14)
Internet	1 (50)	21 (33)	12 (38)	1 (33)	0 (0.0)	0 (0.0)	35 (34)
Information meetings	0 (0.0)	5 (7.9)	0 (0.0)	0 (0.0)	1 (100)	0 (0.0)	6 (5.9)
Other MS patients	0 (0.0)	13 (21)	3 (9.4)	0 (0.0)	0 (0.0)	0 (0.0)	16 (16)
Information not sought	0 (0.0)	12 (19)	2 (6.3)	2 (67)	0 (0.0)	0 (0.0)	16 (16)
Unspecified	0 (0.0)	1 (1.6)	0 (0.0)	0 (0.0)	0 (0.0)	0 (0.0)	1 (1.0)

*n, number; CIS, clinically isolated syndrome; RRMS, relapsing remitting MS; SPMS, secondary progressive MS, DMD, disease modifying drug; im, intramuscular; sc, subcutaneous, IFN, interferon; GA, glatiramer acetate; GP, general practitioner*.

### Role of neurologists in family planning

For all patients (*N* = 271), the frequency at which the topic of pregnancy was addressed by their neurologists was captured by the survey. For 16.6% (45/271) of patients, neurologists initiated the subject of pregnancy at each consultation, whereas 25.5% (69/271) did so sporadically, 27.7% (75/271) did so when the patient asked and 25.8% (70/271) did not raise the topic of pregnancy at all.

### Importance of DMD choice with respect to pregnancy

Therapy choice with respect to family planning was important or very important to 31.4% (85/271) of the total population, compared with 62% (36/58) and 41% (18/44) of patients with a short or medium-term plan to conceive, respectively.

### Pregnancy related concerns

Participants in the survey also provided information about their pregnancy-related concerns. In the total population, 60.9% (165/271) were concerned about the course of the disease after pregnancy, compared with 86.2% (50/58) with a short- and 84.1% (37/44) with a medium-term plan to conceive. The health of the unborn child was an issue for 46.9% (127/271), compared with 69% (40/58) with a short- and 81.8% (36/44) with a medium-term plan to conceive. Taking care of the child after birth mattered to 45% (122/271), compared with 60.3% (35/58) with a short- and 54.5% (24/44) with a medium-term plan to conceive. The course of the disease during pregnancy concerned 43.5% (118/271), compared with 62.1% (36/58) with a short- and 77.3% (34/44) with a medium-term plan to conceive. MS therapy options were an issue for 35.1% (95/271), compared with 44.8% (26/58) with a short- and 63.6% (28/44) with a medium-term plan to conceive. Breast feeding mattered to 21.4% (58/271), compared with 36.2% (21/58) with a short- and 38.6% (17/44) with a medium-term plan to conceive.

### Use of DMD therapy prior to pregnancy

Patient use of DMD therapies prior to planned (*n* = 48) and unplanned (*n* = 9) pregnancies was determined (Table 3). One third of patients with planned pregnancies did not use DMD therapy prior to conceiving (33.3%, 16/48). The most common DMD therapy used prior to planned pregnancies was sc IFN β-1a (31.3%, 15/48). This was followed by natalizumab (10.4%, 5/48), sc IFN β-1b (8.3%, 4/48), fingolimod (6.3%, 3/48), GA (4.2%, 2/48), im IFN β-1a (4.2%, 2/48) and “other” treatments (2.1%, 1/48). In contrast, 88.9% (8/9) of patients with unplanned pregnancies used DMDs. The most commonly used DMDs prior to unplanned pregnancies were fingolimod and GA (22.2%, 2/9), followed by natalizumab, sc IFN β-1a and sc IFN β-1b (11.1%, 1/9). Only patients who were receiving fingolimod (4.2%, 3/71) or natalizumab (2.3%, 2/88) planned to switch therapies due to their plan to conceive.

**Table 3:** Use of DMD prior to pregnancy.

	**Pregnancy status**, ***n***** (%)**			
**Therapy**	**Planned (*n* = 48)**	**Unplanned (*n* = 9)**	**Unspecified (*n* = 2)**	**Total (*n* = 59)**
im IFN β-1a	2 (4.2)	1 (11.1)	0 (0.0)	3 (5.1)
sc IFN β-1a	15 (31.3)	0 (0.0)	1 (50.0)	16 (27.1)
sc IFN β-1b	4 (8.3)	1 (11.1)	0 (0.0)	5 (8.5)
Fingolimod	3 (6.3)	2 (22.2)	0 (0.0)	5 (8.5)
GA	2 (4.2)	2 (22.2)	0 (0.0)	4 (6.8)
Natalizumab	5 (10.4)	1 (11.1)	0 (0.0)	6 (10.2)
“Other”	1 (2.1)	0 (0.0)	0 (0.0)	1 (1.7)
Unspecified	0 (0.0)	1 (11.1)	1 (50.0)	2 (3.4)
No therapy	16 (33.3)	1 (11.1)	0 (0.0)	17 (28.8)

*n, number; im, intramuscular; sc, subcutaneous; IFN, interferon; GA, glatiramer acetate*.

### Use of DMD according to family planning status

**Figure 1:**
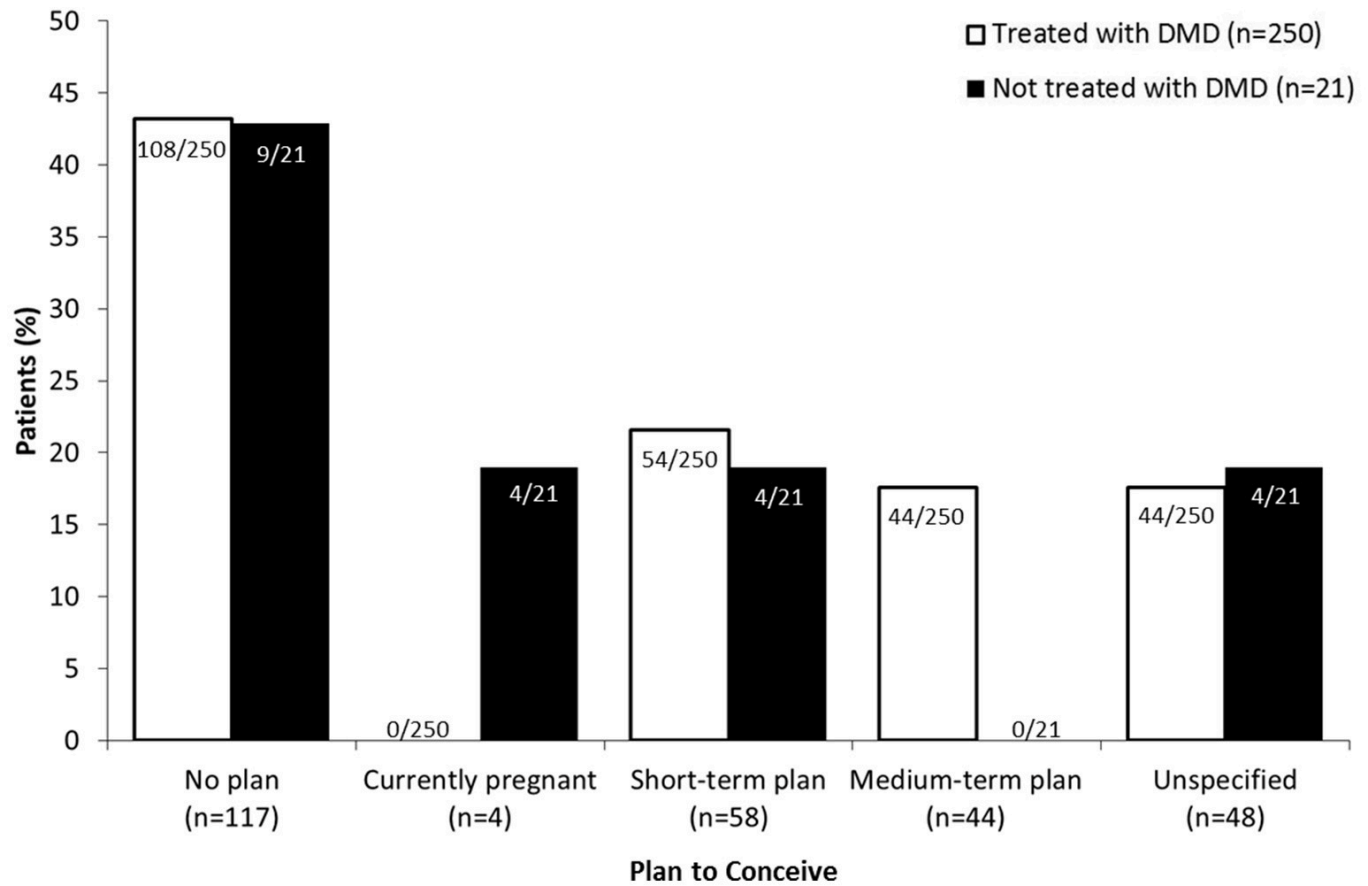
DMD treatment according to plan to conceive. Patients who had no plan to conceive, were pregnant at the time of the survey (“currently pregnant”) or planned to conceive in less (“short-term plan”) or more (“medium-term plan”) than 2 years were asked to specify whether or not they were using DMD (disease modifying drug) therapy. Data for patients planning to conceive are expressed as a percentage of the total number of patients that were treated (white bars; *n* = 250) or not treated (black bars; *n* = 21) with DMDs.

Of those patients being treated with DMDs (92.3%, 250/271), approximately half (43.2%, 108/250) had no plan to conceive, followed by short- or medium-term plan (39.2%, 98/250; Figure 1). None of the participants who were pregnant at the time of the survey (1.5%, 4/271) were being treated with DMDs. Most often, patients did not receive therapy due to child-bearing reasons (42.9%, 9/21); either they wished to conceive (23.8%, 5/21) or were already pregnant (19.0%, 4/21). The remaining patients did not want treatment (28.6%, 6/21) or cited “other” reasons (28.6%, 6/21).

### Type of DMD treatment according to plan to conceive

The type of DMD treatment used by survey participants was evaluated according to their family planning status (Table 4). A significantly higher proportion of women with a short-term plan to conceive (35.2%, 19/54) were treated with injectable therapies compared with those without plans to conceive (17.6%, 19/108; *p* = 0.013 that the two groups are not different). Numerically fewer patients with a medium-term plan to conceive (20.5%, 9/44) were treated with injectables compared with those with a short-term plan (35.2%, 19/54; *p* = 0.108 that the two groups are not different). In addition, the number of women receiving oral treatments was numerically greater in those without plans to conceive (31.5%, 34/108) compared with those with a short-term (24.1%, 13/54) plan. However, women with a medium-term plan (34.1%, 15/44) were proportionally more often treated with oral therapies compared with those without plans to conceive. A higher proportion of women without plans to conceive (38.0%, 41/108) received natalizumab compared with those with a short- (29.6%, 16/54) or medium-term (31.8%, 14/44) plan but this did not reach significance.

**Table 4:** DMD treatment use among female patients according to their plans to conceive.

	**Plan to conceive**				***p*****-value^a^**		
**DMD type^b^**	**No plan (*n* = 108)**	**Short-term^c^ (*n* = 54)**	**Medium-term^d^ (*n* = 44)**	**N/A (*n* = 44)**	**No plan vs. short-term plan**	**No plan vs. medium-term plan**	**Short-term vs. medium-term plan**
Injectable therapies, *n* (%)	19 (17.6)	19 (35.2)	9 (20.5)	7 (15.9)	0.013	0.680	0.108
Oral therapies, *n* (%)	34 (31.5)	13 (24.1)	15 (34.1)	14 (31.8)	0.327	0.755	0.275
Infusion therapy, *n* (%)	41 (38)	16 (29.6)	14 (31.8)	17 (38.6)	0.295	0.475	0.815
Other, *n* (%)	14 (13)	6 (11.1)	6 (13.6)	6 (13.6)	0.736	0.911	0.704

a Pearson's chi-squared test at significance level 0.05, two-sided;

b See Methods for DMD groups;

c < 2 years;

d *≥2 years*.

The Swiss SmPC for each DMD gives the following recommendations for women of childbearing age:(23) IFN β/glatiramer acetate/DMF, use a reliable contraception method; natalizumab, if pregnancy occurs, therapy cessation should be considered; fingolimod, use a reliable contraception method during therapy and within 2 months of therapy cessation; teriflunomide, use a reliable contraception method and must not be applied in pregnancy (can only become pregnant if teriflunomide blood concentration is < 0.02 mg/L).

*DMD, disease modifying drug; DMF, dimethyl fumarate; n, number; N/A, not applicable*.

### Plan to conceive and DMD treatment in accordance with age

The majority of participants under 20 years of age (66.7%, 2/3) had a medium-term plan to conceive, whilst those aged 20–30 years mostly had a medium- (48.1%, 37/77) or short-term (33.8%, 26/77) plan to conceive (Table 5). However, the majority of all other participants, aged over 31 years, had no plans to conceive (31–40 years, 43.3%, 42/97; 41–45 years, 76.3%, 29/38; >45 years, 61.5%, 32/52). Overall, participants mostly (60.5%, 164/271) stated that treatment with respect to pregnancy was not important. This was also true for individual age categories, where treatment with respect to pregnancy was not important to greater than 50% of the populations. More specifically, treatment with respect to pregnancy was very important or important for those aged 20–30 years, 45.5%; 31–40 years, 37.1%; 41–45 years, 7.9%; and >45 years, 13.5% (Table 5).

**Table 5:** Plan to conceive and DMD treatment in accordance to age.

	**Age, years**, ***n***** (%)**						
	** < 20**	**20–30**	**31–40**	**41–45**	**>45**	**Unspecified**	**Total**
	***n* = 3**	***n* = 77**	***n* = 97**	***n* = 38**	***n* = 52**	***n* = 4**	***N* = 271**
**PLAN TO CONCEIVE**							
No plan	0 (0.0)	11 (14.3)	42 (43.3)	29 (76.3)	32 (61.5)	3 (75.0)	117 (43.2)
Currently pregnant	0 (0.0)	3 (3.9)	0 (0.0)	1 (2.6)	0 (0.0)	0 (0.0)	4 (1.5)
Short-term	0 (0.0)	26 (33.8)	28 (28.9)	3 (7.9)	0 (0.0)	1 (25.0)	58 (21.4)
Medium-term	2 (66.7)	37 (48.1)	4 (4.1)	0 (0.0)	1 (1.9)	0 (0.0)	44 (16.2)
Unspecified	1 (33.3)	0 (0.0)	23 (23.7)	5 (13.2)	19 (36.5)	0 (0.0)	48 (17.7)
**DMD TREATMENT**							
Yes	3 (100)	72 (93.5)	91 (93.8)	34 (89.5)	47 (90.4)	3 (75.0)	250 (92.3)
No	0 (0.0)	5 (6.5)	6 (6.2)	4 (10.5)	5 (9.6)	1 (25.0)	21 (7.7)
**IMPORTANCE OF TREATMENT IN RESPECT TO PREGNANCY**							
(Very) important	1 (33.3)	35 (45.5)	36 (37.1)	3 (7.9)	7 (13.5)	3 (75.0)	85 (31.4)
Not important	2 (66.7)	41 (53.2)	54 (55.7)	31 (81.6)	35 (67.3)	1 (25.0)	164 (60.5)
Not specified	0 (0.0)	1 (1.3)	7 (7.2)	4 (10.5)	10 (19.2)	0 (0.0)	22 (8.1)

*n, number; DMD, disease modifying drug*.

### Treatment recommendations and time after stopping DMD until conception in the total population

In total, 28.0% of patients (76/271) stated that their neurologists made suggestions regarding DMD therapy based on their plans to conceive at some point during the disease course. Within this group, injectables were recommended to 48.7% (37/76; including 28.9% sc IFN β-1a and 23.7% GA), natalizumab to 11.8% (9/76), fingolimod to 5.3% (4/76) and “other” treatments were recommended to 2.6% (2/76). In addition, 55.3% of patients (42/76) stated that their neurologists recommended they should not use fingolimod. They also advised 18.4% (14/76) of patients not to use teriflunomide, and 17.1% (13/76) not to use natalizumab, 10.5% (8/76) not to use injectables and 9.2% (7/76) not to use “other” treatments.

Since being diagnosed with MS, 15.5% (42/271) of the participants had one child, 21.8% (59/271) had one or more and 6.3% (17/271) had 2 or more. In addition, 66.4% (180/271) of participants had no children since MS diagnosis. Of the 59 women with pregnancies, 9 (15.3%) stated that their last pregnancy was unplanned. Prior to their last pregnancy, 13.6% (8/59) of patients stopped treatment and contraception at the same time, 18.6% (11/59) stopped treatment but continued to use contraception for a period of time afterwards, and 37.3% (22/59), discontinued DMD treatment when pregnancy was detected. For the remaining patients (18/59, 30.5%) no information was given on treatment discontinuation.

**Figure 2:**
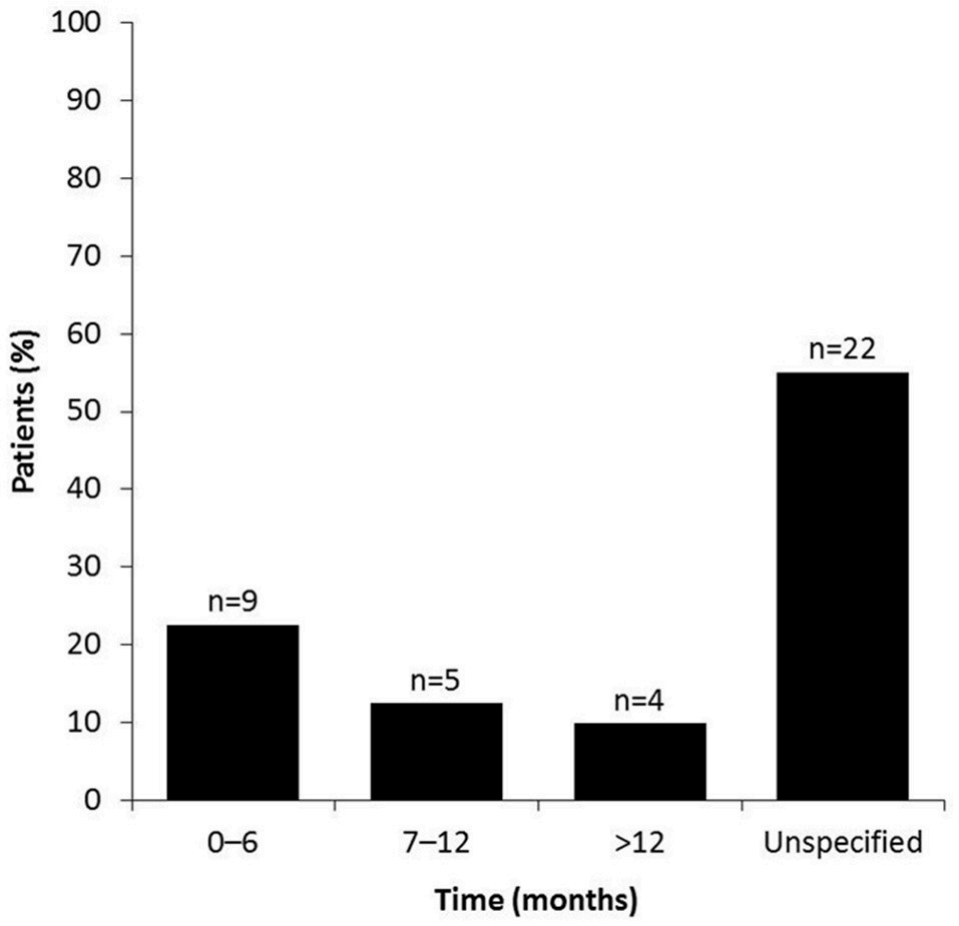
Time between stopping MS therapy and conception. Survey participants were asked to specify the duration of time (0–6, 7–12, or >12 months) taken between stopping DMD treatment and terminating the use of conception, with a view to conceive. Patients who did not give an answer were placed in the “unspecified” group.

For women with short-term plans to conceive, the time period between stopping MS therapy and conception ranged from 0 to 6 months in 22.5% (9/40), 7 to 12 months in 12.5% (5/40), and >12 month in 10% (4/40) of patients (Figure 2). Over half of the women (55%, 22/40) did not specify the time taken between stopping DMD therapy for MS and conception for unknown reasons.

## Discussion

Over one third of participants from the Swiss Women with MS patient survey were pregnant or planned to conceive and nearly all received DMD treatment independent of their family planning status. Family planning was amongst the most important factors in context of the disease, and not surprisingly, more important to women with plans to conceive compared with the whole population. Major pregnancy-related concerns included the disease course during and after pregnancy, the health of the child and treatment options before and during pregnancy. This, amongst others reasons, is due to the fact that limited information is currently available on the safety of DMD use during pregnancy and therefore, pregnancy usually leads to an inadvertent treatment break.

Therapy choice with respect to pregnancy was important or very important to the majority of women with a short-term plan to conceive. For these patients, a numerically higher proportion used injectable DMDs compared with oral and infusion therapies. In addition, significantly more women with a short-term plan used injectables than those without a plan to conceive. Overall, injectable DMDs were the most commonly used therapies prior to almost half of all recorded planned pregnancies, with sc IFN β-1a being the predominantly used DMD by approximately one third of patients. A further one third of patients with planned pregnancies did not use DMD treatment and no patients who were pregnant at the time of the survey were using DMD therapy.

Oral therapies (fingolimod and teriflunomide) and natalizumab were proportionally used more often (not statistically significant) by women without plans to conceive than by those with a short- or medium-term plan (natalizumab only). Of interest, only patients who were treated with fingolimod or natalizumab planned to switch therapies due to planned pregnancy.

The predominant use of injectable DMDs prior to planned pregnancies can likely be attributed to the long-term experience and studies which have shown that injectable DMDs do not substantially affect pregnancy outcomes (6, 7). In contrast, the data on the potential harmful effects of natalizumab and oral DMDs, in particular fingolimod, explains the restricted use of these drugs in the context of planned pregnancy (13, 14).

Almost half (49%) of all pregnancies in the USA are unplanned (21) and given that approximately 15% of pregnancies in this study were unplanned, the risk of using potentially teratogenic drugs during early pregnancy should be considered in women with MS of child-bearing age. In addition, there was often a time-lapse between stopping DMD therapy and conception of more than 6 months. Treatments that have no influence on pregnancy outcomes would be of major advantage in such cases, since unplanned pregnancies would not be jeopardised and treatment gaps lasting uncertain periods of time before conception could be avoided. These factors may provide further reasons for the treatment choices observed in this study.

Overall, approximately one third of patients who took part in the survey were taking natalizumab. This was because the majority of questionnaires were distributed in a hospital setting. However, numerically more women without a plan to conceive used natalizumab compared with those with a short- or medium-term plan. No patients in this study were treated with DMF, which reflects the fact that it was only granted authorisation for use in Switzerland near the beginning of the study (August 2014).

Less than half of patients surveyed reported that the topic of pregnancy was raised by their neurologist during previous consultations, with less than one fifth indicating that their neurologists addressed the issue at every visit. This may explain the fact that women with plans to conceive often sought information regarding treatment options and family planning from alternative sources, including the internet, information meetings and other MS patients. However, discussing treatment options in regard to pregnancy should be guided by the treating neurologist since they have a greater understanding of the MS treatment options available and their respective safety profiles.

## Study limitations

In order to examine a population that was representative of female Swiss MS patients, the questionnaire was distributed by 15 Swiss MS centres, 8 of which were hospital-based and 7 were private practices. However, given that the questionnaire was not validated and a limited number of patients participated in the study, with the overall response rate unknown, it is possible that a subset of patients with a special interest in the topic of family planning may have responded to the questionnaire. This remains unclear due to the lack of epidemiological data concerning this matter in Switzerland.

## Conclusion

This was the first study to evaluate the role of family planning in the selection of DMD therapy amongst women with MS, and the influencing factors that affect therapy choice. The study highlights that pregnancy remains an important yet unresolved concern in the treatment of MS patients. This leads to uncertainty with regard to disease course and the health of the unborn child. Family planning influences treatment decisions and in most cases, results in a therapy pause. Since injectables can be administered until pregnancy occurs (22), the use of these therapies prevents the need for a therapy pause before conception.

Taken together, the results of this study provide an understanding of some factors affecting women with MS planning a family in order to guide treatment recommendation.

## Ethics statement

Correspondence with the Ethics Committee Bern (Switzerland) concluded that Ethical Committee (EC) approval was not required for the Women with Multiple Sclerosis Patient Survey, as the patient surveys were conducted anonymously.

## Author contributions

All authors made substantial contributions to the concept and design, or analysis and interpretation of data, and to the drafting of the manuscript or revising it critically for important intellectual content. In addition, all authors provided final approval of the manuscript.

### Conflict of interest statement

SMuehl and DM are employees of Merck (Schweiz) AG, an affiliate of Merck KGaA, Darmstadt, Germany. SMüller received honoraria for travel, honoraria for lectures/consulting and/or grants for studies from Biogen Idec, Novartis, Teva, Merck, Genzyme and Bayer Schweiz AG. AC served as advisor and received speaker compensations from Allergan, Bayer, Biogen, Genzyme, Merck Serono, Novartis and Teva. LA served as advisor and received speaker and travel compensations from Bayer, Biogen Idec, Genzyme, Merck Serono, Novartis and Teva. PS has received honoraria as advisor/speaker from Merck, Biogen Idec, Genzyme, Teva and Novartis. CK has received honoraria for lectures as well as research support from Biogen, Novartis, Almirall, Bayer Schweiz AG, Teva, Merck, Genzyme, Roche and the Swiss MS Society (SMSG). GDV received honoraria for travel, lectures/consulting from Biogen Idec, Genzyme, Merck, Novartis, Roche, Teva.
