# Supplementary material for: Role of Family Planning in Women With Multiple Sclerosis in Switzerland: Results of the Women With Multiple Sclerosis Patient Survey
Source: Front Neurol. 2018 Oct 10;9:821. doi: 10.3389/fneur.2018.00821 (PMC6191482; doi:10.3389/fneur.2018.00821)
Supplement: Supplementary file 1 [file Data_Sheet_1.docx]

**Multiple Sclerosis and Family Planning -**

**A Questionnaire for Female MS Patients**

Multiple sclerosis (MS) is generally diagnosed between 20 and 40 years of age and more frequently in women than in men. This means that many MS patients are women of childbearing age who potentially wish to conceive.

The purpose of this anonymous questionnaire is to examine, as a collaborative project between the neurology outpatients department at Bern University Hospital (Prof. Dr. med. H. Mattle and Dr. med. C. Kamm) and Merck Serono, what role the subject of family planning plays in your life, what matters to you about it and how this subject is addressed in the context of your treatment.

The results of this survey are intended to be used to improve understanding of the factors which affect women with MS in this context so that the treatment of MS can be optimised as much as possible.

You have two ways of taking part in the survey: you can complete the questionnaire either on paper or online. In both cases, the data collected is completely anonymous. The data will be analysed jointly with the agency brunner & hess from Zurich.

**Completing the questionnaire on paper:**

Please send the completed questionnaire in the return envelope supplied to:

*brunner & hess software ag*

*Hoffnungsstrasse 3*

*8038 Zurich*

Please do not put your sender's address on the return envelope.

**Completing the questionnaire online:**

To do so, please go to [**www.womenwithms.ch**](http://www.womenwithms.ch) and enter your access code.

You will find your access code in the header of this document.

**Instructions for the questionnaire**

This questionnaire consists of 26 questions subdivided into the following 3 subject sets:

I: General questions (questions 1 - 7)

II: Questions about your situation (questions 8 - 18)

III: Questions about pregnancies (questions 19 - 26)

When answering questions about previous pregnancies, please always refer to your last pregnancy.

Your neurologist has given you the questionnaire in an envelope.

Question No. 2 from the questionnaire "What form of MS do you have?" is printed on the envelope. Your neurologist has already answered this question for you.

Please use your neurologist's answer when completing the questionnaire.

**Subject set I: General questions**

**Question 1:** What age group are you in?

|  |  |  |
| --- | --- | --- |
|  |  | under 20 years old |
|  |  | 20 - 30 years old |
|  |  | 31 - 40 years old |
|  |  | 41 - 45 years old |
|  |  | over 45 years old |

**Question 2:** What form of MS do you have?

(Your neurologist has answered this question on the survey's envelope. Please use this answer)

|  |  |  |
| --- | --- | --- |
|  |  | CIS (clinically isolated syndrome) |
|  |  | RRMS (relapsing-remitting MS) |
|  |  | SPMS (secondary progressive MS) |

**Question 3:** How long ago were you diagnosed?

|  |  |  |
| --- | --- | --- |
|  |  | 0 - 3 years |
|  |  | 3 - 5 years |
|  |  | 5 - 10 years |
|  |  | over 10 years |

**Question 4:** Is your neurologist male or female?

|  |  |  |
| --- | --- | --- |
|  |  | female |
|  |  | male |
|  |  | it varies |

**Question 5:** How important to you are the following points in the context of the disease?

(1: not very important - 5: very important)

|  |  |  |  |  |  |
| --- | --- | --- | --- | --- | --- |
|  | not very important  1 | 2 | 3 | 4 | very important  5 |
| Being relapse-free: |  |  |  |  |  |
| Delaying the worsening of disability: |  |  |  |  |  |
| Children/family planning: |  |  |  |  |  |
| Work: |  |  |  |  |  |
| Relationship: |  |  |  |  |  |
| Independence: |  |  |  |  |  |

**Question 6:** What questions concern you about the subject of MS and having children? (Select all that apply)

|  |  |  |
| --- | --- | --- |
|  |  | Disease course during pregnancy |
|  |  | Disease course after pregnancy |
|  |  | Health of the (unborn) child |
|  |  | Coping with the child after birth (handling additional demands) |
|  |  | Breastfeeding |
|  |  | MS treatment options |
|  |  | Don't know |

**Question 7:** Where have you obtained information about the various long-term MS treatment options for patients wishing to conceive?

(Select all that apply)

|  |  |  |
| --- | --- | --- |
|  |  | My neurologist |
|  |  | Family doctor |
|  |  | MS nurse |
|  |  | Gynaecologist |
|  |  | Internet |
|  |  | Information events on the subject |
|  |  | Interactions with other people with MS |
|  |  | I have not obtained any information on this subject |
|  |  | Don't know |

**Subject set II: Questions about your situation**

**Question 8:** Would you like to conceive? (Select all that apply)

|  |  |  |
| --- | --- | --- |
|  |  | I am pregnant at the moment |
|  |  | Yes, I would like to conceive in the near future |
|  |  | Yes, I would like to become pregnant within the next two years |
|  |  | Yes, but not for 2 years or more |
|  |  | No, because of my MS |
|  |  | No |
|  |  | Don't know |

**Question 9:** Are you receiving long-term MS treatment?

|  |  |  |
| --- | --- | --- |
|  |  | Yes (Please go straight to question 10) |
|  |  | No (Please go straight to question 11) |

**Question 10:** Which long-term MS treatment are you currently receiving?

|  |  |  |
| --- | --- | --- |
|  |  | Aubagio |
|  |  | Avonex |
|  |  | Betaferon |
|  |  | Copaxone |
|  |  | Gilenya |
|  |  | Rebif |
|  |  | Tysabri |
|  |  | Other |

(Please go straight to question 12)

**Question 11:** What is the main reason why you are not receiving long-term MS treatment?

|  |  |  |
| --- | --- | --- |
|  |  | I am having a break from treatment because I wish to conceive |
|  |  | I have stopped taking treatment |
|  |  | I do not want any treatment |
|  |  | Other reasons |

**Question 12:** How important was the subject of pregnancy/family planning in your current choice of treatment?

|  |  |  |
| --- | --- | --- |
|  |  | Not important |
|  |  | Important |
|  |  | Very important |

**Question 13:** Have you switched treatment in the past because of your wish to conceive?

|  |  |  |
| --- | --- | --- |
|  |  | Yes (Please go straight to question 14) |
|  |  | No (Please go straight to question 15) |

**Question 14:** You have switched treatment in the past because of your wish to conceive. From which to which treatment did you switch?

|  |  |  |
| --- | --- | --- |
|  | From | To |
| Aubagio |  |  |
| Avonex |  |  |
| Betaferon |  |  |
| Copaxone |  |  |
| Gilenya |  |  |
| Rebif |  |  |
| Tysabri |  |  |
| Other |  |  |

**Question 15:** Are you currently planning to switch treatment because of your wish to conceive?

|  |  |  |
| --- | --- | --- |
|  |  | Yes |
|  |  | No |

**Question 16:** How often does your neurologist bring up the subject of conceiving/family planning?

|  |  |  |
| --- | --- | --- |
|  |  | At every check-up |
|  |  | Sporadically |
|  |  | Only when I bring up the subject |
|  |  | Never |

**Question 17:** Has your neurologist ever advised you to take a particular MS treatment or not to take any other MS treatment(s) because of your wish to conceive?

|  |  |  |
| --- | --- | --- |
|  |  | Yes |
|  |  | No (please go straight to question 19) |

**Question 18:** You selected "Yes" in question 17. What treatment did your neurologist advise you to take or not to take because of your wish to conceive? (Select all that apply)

|  |  |  |
| --- | --- | --- |
|  | Advised to take | Advised not to take |
| Aubagio |  |  |
| Avonex |  |  |
| Betaferon |  |  |
| Copaxone |  |  |
| Gilenya |  |  |
| Rebif |  |  |
| Tysabri |  |  |
| Other |  |  |

**Subject set III: Questions about previous pregnancies**

When answering questions about previous pregnancies, please always refer to your last pregnancy.

**Question 19:** Have you had a child/children since your CIS/MS diagnosis? If yes, how many?

|  |  |  |  |
| --- | --- | --- | --- |
|  |  | Yes, one child | (please go straight to question 20) |
|  |  | Yes, 2 or more children | (please go straight to question 20) |
|  |  | No | (please go straight to question 25) |

**Question 20:** How long ago was your youngest child born?

|  |  |  |
| --- | --- | --- |
|  |  | I am pregnant at the moment |
|  |  | 0 - 1 year |
|  |  | between 1 - 2 years |
|  |  | between 2 - 3 years |
|  |  | more than 3 years |

**Question 21:** Was your last pregnancy planned or unplanned?

|  |  |  |
| --- | --- | --- |
|  |  | planned |
|  |  | unplanned |
|  |  | Don't know |

**Question 22:** What was your last MS treatment before your last pregnancy began?

|  |  |  |
| --- | --- | --- |
|  |  | No treatment |
|  |  | Aubagio |
|  |  | Avonex |
|  |  | Betaferon |
|  |  | Copaxone |
|  |  | Gilenya |
|  |  | Rebif |
|  |  | Tysabri |
|  |  | Other |

**Question 23:** What was your approach to conceiving in your last pregnancy?

|  |  |  |  |
| --- | --- | --- | --- |
|  |  | I stopped taking the treatment and using contraception at the same time  (go straight to question 24) |  |
|  |  | I stopped taking the treatment and carried on using contraception for a while  (go straight to question 24) |  |
|  |  | I stopped taking the treatment when my pregnancy test result was positive  (go straight to question 25) |  |

**Question 24:** You stopped taking your long-term MS treatment because of your wish to conceive. How long did it take for you to become pregnant after you stopped taking the MS treatment?

|  |  |  |
| --- | --- | --- |
|  |  | 0 - 6 months |
|  |  | 7 - 12 months |
|  |  | over 12 months |
|  |  | Don't know |

**Question 25:** What approach are you planning to adopt for a future pregnancy?

|  |  |  |
| --- | --- | --- |
|  |  | I will stop taking the treatment and using contraception at the same time |
|  |  | I will stop taking the treatment and carry on using contraception for a while |
|  |  | I will stop taking the treatment when my pregnancy test result is positive |
|  |  | Don't know |

**Question 26:** What is your current approach to contraception?

|  |  |  |
| --- | --- | --- |
|  |  | I use contraception carefully |
|  |  | I do not use contraception |
|  |  | Don't know |

Thank you very much for answering this questionnaire.

**If you have answered the questionnaire on paper:**

Please send the completed questionnaire anonymously in the return envelope supplied to:

*brunner & hess software ag*

*Hoffnungsstrasse 3*

*8038 Zurich*
